# Development of a Direct Headspace Collection Method from *Arabidopsis* Seedlings Using HS-SPME-GC-TOF-MS Analysis

**DOI:** 10.3390/metabo3020223

**Published:** 2013-04-09

**Authors:** Miyako Kusano, Yumiko Iizuka, Makoto Kobayashi, Atsushi Fukushima, Kazuki Saito

**Affiliations:** 1 RIKEN Plant Science Center, Tsurumi, Yokohama 230-0045, Japan; E-Mails: kobamako@riken.jp (M. Kobayashi); a-fukush@psc.riken.jp (A.F.); 2 Department of Genome System Sciences, Graduate School of Nanobioscience, KIHARA Institute for Biological Research, Yokohama City University, Totsuka, Yokohama 244-0813, Japan; E-Mail: mkusano005@psc.riken.jp (M.K.); 3 Department of Genome System Science, Graduate School of Nanobioscience, Yokohama city University, Kanazawa, Yokohama 236-0027, Japan; E-Mail: yiizuka-ri@psc.riken.jp (Y.I.); 4 Department of Molecular Biology and Biotechnology, Graduate School of Pharmaceutical Science, Chiba University, Chuo, Chiba 260-8675, Japan; E-mail: ksaito@psc.riken.jp (K.S.)

**Keywords:** solid-phase microextraction, HS-SPME-GC-TOF-MS, volatile organic compounds, VOC profiling, *Arabidopsis*

## Abstract

Plants produce various volatile organic compounds (VOCs), which are thought to be a crucial factor in their interactions with harmful insects, plants and animals. Composition of VOCs may differ when plants are grown under different nutrient conditions, *i.e.*, macronutrient-deficient conditions. However, in plants, relationships between macronutrient assimilation and VOC composition remain unclear. In order to identify the kinds of VOCs that can be emitted when plants are grown under various environmental conditions, we established a conventional method for VOC profiling in *Arabidopsis thaliana* (*Arabidopsis*) involving headspace-solid-phase microextraction-gas chromatography-time-of-flight-mass spectrometry (HS-SPME-GC-TOF-MS). We grew *Arabidopsis* seedlings in an HS vial to directly perform HS analysis. To maximize the analytical performance of VOCs, we optimized the extraction method and the analytical conditions of HP-SPME-GC-TOF-MS. Using the optimized method, we conducted VOC profiling of *Arabidopsis* seedlings, which were grown under two different nutrition conditions, nutrition-rich and nutrition-deficient conditions. The VOC profiles clearly showed a distinct pattern with respect to each condition. This study suggests that HS-SPME-GC-TOF-MS analysis has immense potential to detect changes in the levels of VOCs in not only *Arabidopsis*, but other plants grown under various environmental conditions.

## 1. Introduction

Plants and animals, including humans and microorganisms, can produce volatile organic compounds (VOCs). For example, VOCs in exhaled breath show specific profile patterns with respect to various cancers [1,2,3,4,5,6,7,8]. *In vitro* and *in vivo* experiments have been conducted for VOC profiling in rat and cattle breath as case studies of animal VOCs [9,10]. Microorganisms can produce various types of VOCs, which are likely to have crucial effects on soil ecology [11] and on interactions across other organisms [12,13,14,15]. Plants also contain a large variety of VOCs, including simple gases and other VOCs. At present, approximately 1,700 compounds are identified as VOCs, and these account for more than 1% of plant secondary metabolites [16,17]. The main classes of plant VOCs are terpenoids, phenylpropanoids/benzenoids and fatty acid and amino acid derivatives [18]. Plants can emit various types of VOCs, not only to communicate with herbivores, microbes and neighboring plants, but also to respond to abiotic factors, such as light and/or temperature, atmospheric CO_2_ concentration or nutrition [19,20,21]. Therefore, plant VOCs are potential biomarkers capable of identifying the physiological states of plants and the stresses to which they have been subjected.

Sensitive methods have been developed for headspace (HS) sampling and VOC analysis. HS sampling is a non-destructive method for collecting volatile compounds. Static and dynamic sampling methods are widely used for collecting HS samples [22]. Static HS sampling is suited for qualitative analyses of VOCs and surveys of VOC profiles of different plant species or cultivars at a single time point, while dynamic HS sampling enables us to quantify changing VOC emissions in the form of real-time analysis. Many devices can be used to collect VOCs, e.g., solid phase microextraction (SPME) fibers, stir bar sportive extraction (SBSE) devices [23,24] and in-tube extraction (ITEX) [25,26]. Sensor arrays composed of thin nanostructure films, such as monolayer-capped metallic nanoparticles and chemiresistive random network of single-walled carbon nanotubes, can be used as diagnostic tools to detect VOCs in exhaled breath samples [27,28]. 

Gas chromatography (GC)-based techniques are widely used for static HS analyses, including GC-mass spectrometry (GC-MS), GC-flame ionization detection (GC-FID), GC-electroantennogram detection (GC-EAD) and two-dimensional GC-MS (GC×GC-MS) [17,22,29]. We can directly inject a mixture of VOCs into GC instruments, since no derivatization steps are required. Fast isoprene sensor chemiluminescence detection (FIS), laser-based infrared photoacoustic (PA) spectroscopy and proton transfer reaction–mass spectrometry (PTR-MS) enabled us to conduct real-time detection of VOCs from plants [18]. 

One ultimate goal of metabolomics is to identify and quantify the metabolome in organisms, which includes VOCs. Development of MS-based technologies in metabolomics enables the measurement of hundreds of metabolites [30,31,32]. In particular, electron impact-time-of-flight-MS (EI-TOF-MS) provides us with comprehensive information on molecular fragments as mass-to-charge ratios [33]. Annotation of each spectrum of a VOC is performed by finding an exact match to a reference spectrum from well-established libraries, such as NIST/EPA/NIH Mass Spectral Library [34], Adams Library [35], Terpenoids Library (http://massfinder.com/wiki/Terpenoids_Library) and VocBinbase [36]. The Kovats retention index (RI), calculated by comparing the chromatographic retention time of *n*-alkane peaks, is often used to annotate detected peaks [37]. A combination of these libraries and RI information is indispensable to annotate detected peaks in a non-targeted matter. So far, HS-SPME-GC-MS analysis has been applied to the comprehensive VOC profiling of apple fruits [38], tomato flavors [39,40], human nevi and melanoma [6] and fungus extracts [41].

The VOCs emitted from the flowers and foliage of *Arabidopsis thaliana* (*Arabidopsis*), which is widely used as a model species for studying plant biology, have been investigated [42,43,44,45]. However, many studies were focused on the VOCs induced by attacking herbivores [46,47,48] and microorganisms [49,50]. We hypothesized that the composition of VOCs may differ when grown under different nutrient conditions, *i.e.*, macronutrient-deficient conditions. However, the relationships in the plants between macronutrient assimilation, metabolic networks and VOC composition in these plants remain unclear. 

**Figure 1:**
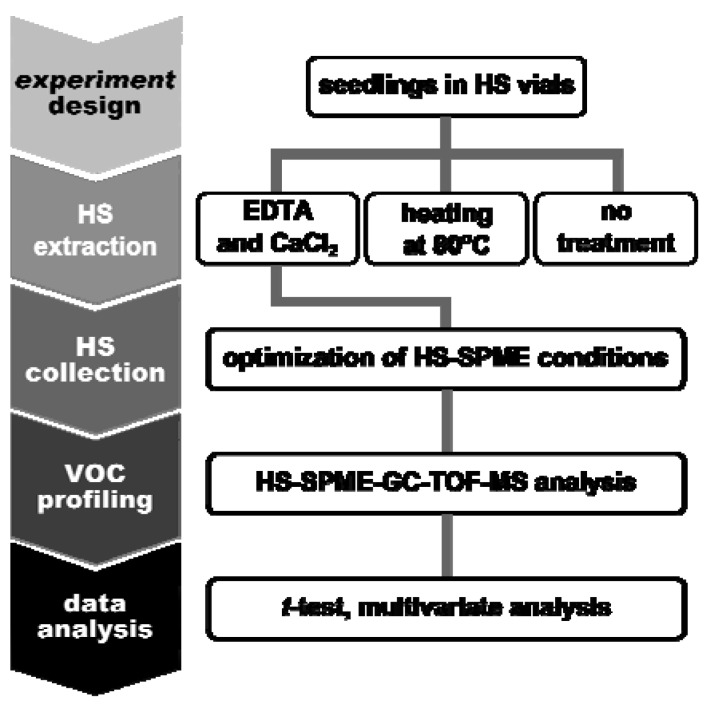
Volatile organic compound (VOC) profiling of *Arabidopsis* seedlings directly in headspace (HS) vials. Abbreviation: EDTA, ethylene-diamine-tetra-acetic acid.

To obtain insights into these relationships, we developed a way of VOC profiling by using HS-SPME-GC-TOF-MS (Figure 1). First, *Arabidopsis* seedlings were directly grown in HS vials. Volatile compounds in each vial were collected using an appropriate SMPE fiber and analyzed by using HS-SPME-GC-TOF-MS in a non-targeted manner. After data processing and normalization as described by Kusano *et al.* [51], we conducted statistical analysis on the dataset. We performed VOC profiling in the HS of *Arabidopsis* seedlings grown under two nutrient conditions as a case example. Distinct patterns of VOC profiles were found by applying the optimized analytical method. The annotated VOCs were determined by performing multivariate analysis and *t*-tests as discriminative parameters between the two conditions.

## 2. Results and Discussion

### 2.1. Experiment Design for VOC Profiling in the HS of Arabidopsis Seedlings

**Figure 2:**
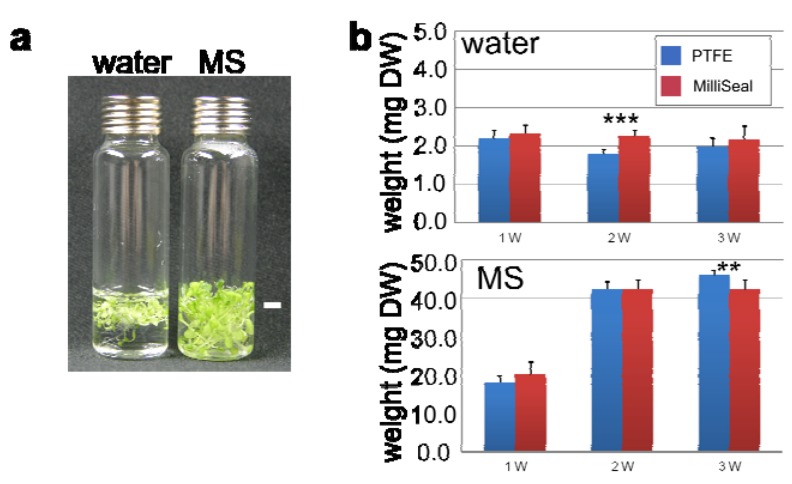
Biomass of *Arabidopsis* seedlings grown under the developed cultivation system. **(a)** Visual phenotypes of *Arabidopsis* seedlings grown in sterilized Milli-Q water (left) or sterile liquid culture in Murashige and Skoog (MS) medium and 1% sucrose (right) after seven-day cultivation. Twenty seedlings were put in an HS vial. Scale bar, 5cm. **(b)** Biomass of *Arabidopsis* seedlings grown in vials sealed by a silicon/polytetrafluoroethylene septa (PTFE) cap and a MilliSeal cap (*n* = 10 for each condition). Significance was determined by *t*-test (*p*-value: ** *p* < 0.005; *** *p* < 0.0005). Abbreviations: DW, dry weight; W, week.

Before starting the experiment, we performed HS-SPME-GC-TOF-MS to confirm that the growth medium and water for cultivating *Arabidopsis* seedlings contained no volatile substances (data not shown). We grew *Arabidopsis* seedlings in a closed HS vial in order to conduct HS analysis directly (Figure 1a). *Arabidopsis* seedlings in sterile liquid culture containing Murashige and Skoog medium with 1% sucrose (MS condition, hereafter) grew better compared to those in sterilized Milli-Q water (water condition, hereafter, Figure 2a). Seedlings grew 10–20 times larger under the MS condition than water, in terms of dry weight (Figure 2b, blue bar). We next evaluated the effect on CO_2_ content toward plant growth (CO_2_ depletion inhibits plant growth) by weighing the dry matter from *Arabidopsis* seedlings grown in an HS vial, which was closed with a magnetic screw cap equipped with silicon/polytetrafluoroethylene septa (PTFE) or affixed with MilliSeals. There were no drastic effects toward growth whether we used PTFE or MilliSeal seals (Figure 2b). Thus, we expected that changes in the VOC levels from samples could be captured using the proposed growth system. This kind of growth system has been used to investigate short- and long-term responses to nitrogen depletion and nitrate re-addition in *Arabidopsis* [52]. 

### 2.2. Optimization of HS Collection for VOC Profiling

VOCs in the HS of *Arabidopsis* seedlings could be collected directly by transferring the HS vials to the autosampler. To optimize extraction and collection of the HS grown in water and MS conditions, we first used three extraction methods (Figure 1). Next, we used nine fibers to evaluate the collection efficiency of the VOCs. By using the selected fiber, we optimized incubation conditions by changing the incubation time and temperature to collect the HS from *Arabidopsis* seedlings.

#### 2.2.1. HS Extraction

Since the seedlings were alive in each vial just before HS collection, it was necessary to inactivate certain enzymes, such as lipoxygenase and hydroperoxide lyases, which contribute to VOC production. We compared each total ion chromatogram (TIC) obtained by applying the three extraction methods for the water and MS samples. CaCl_2_ is commonly used to stop enzyme activity, as well as to promote the release of VOCs to the HS from the plant tissues [53]. Ethylene-diamine-tetra-acetic acid (EDTA) can also inhibit enzyme activity in the cell [54,55]. In addition, the combined use of EDTA and CaCl_2_ provides highly reproducible VOC emission from tomato fruit [39], which is important when conducting metabolomic analysis. Therefore, we added EDTA, followed by CaCl_2_, to each HS vial for HS extraction. To validate the HS extraction method using EDTA and CaCl_2_, we heated other sets of HS vials to 80 °C to quench the enzyme activity in the seedlings. HS-VOCs emitted from untreated seedlings were also collected. We used *Arabidopsis* seedlings grown in water and MS conditions and performed HS-SPME-GC-TOF-MS analysis on these samples. A distinct pattern was seen in TICs of HS-VOCs, which were extracted by (i) adding EDTA solution followed by CaCl_2_ and (ii) heating at 80 °C. TICs of HS-VOCs from treatment (ii) and those emitted from the untreated seedlings looked similar, particularly when the samples were grown in water (Supplementary Figure S1). Peaks detected in samples from treatment (i) outnumbered other methods (Supplementary Figure S2). On the basis of the results, we chose to use a combination of EDTA and CaCl_2_ to extract HS-VOCs for further analysis.

#### 2.2.2. SPME Fiber Selection

**Figure 3:**
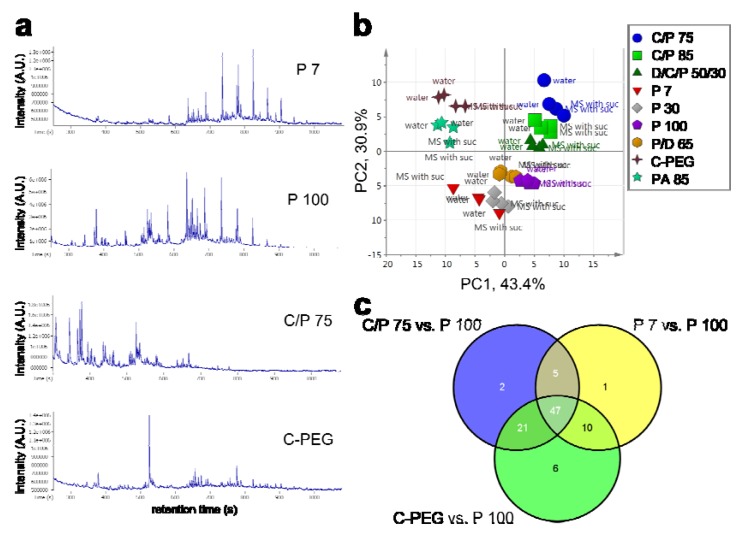
Collection efficiency of HS-VOCs by using various solid phase microextraction (SPME) fibers. **(a)** Total ion chromatograms (TICs) of VOC profiles collected by using a 7-μm polydimethylsiloxane (PDMS) fiber (P 7), a 100-μm PDMS fiber (P 100), a 75-μm carboxen/PDMS fiber (C/P 75) and a carbowax-polyethylene glycol fiber (C-PEG). X-axis represents retention time, while the Y-axis shows peak intensity. **(b)** Principal component analysis (PCA) score scatter plot of VOC profiles using nine SPME fibers (observation: samples, *n* = 36; variables: annotated peaks, *n* = 99). Four independent samples were analyzed using each SMPE fiber. HS-VOCs were collected from *Arabidopsis* seedlings grown in water and MS conditions (*n* = 2 each). **(c)** Venn diagram of the annotated peaks that showed significant changes in the profiles of (i) the 75-μm C/P (blue), (ii) the 7-μm PDMS (yellow) and (iii) the C-PEG fiber collection (green) when compared to those collected by the 100-μm PDMS fiber. The number in the blue circle represents how many annotated peaks showed significant changes in the profiles from the HS-GC-TOF-MS analysis by using the 75-μm C/P fiber. The number in the yellow circle represents the number of significant peaks in the profile from the 7-μm PDMS analysis. The number in the green circle represents the number of significant peaks in the HS profile from the C-PEG fiber collection. Control, the HS profile collected by the 100-μm PDMS fiber (see Table 1). Abbreviation: PC, principal component.

Many types of SPME fibers are available, which is one of the advantages of choosing the SPME method to collect the HS. According to the molecular weight and polarity of the analytes, an appropriate fiber should be chosen. We tested nine fibers to collect the HS from seedlings grown under the two conditions (Figure 2). Figure 3a presents representative TICs of HS-VOCs collected by using a 7-μm-thick layer of polydimethylsiloxane (PDMS), 100-μm PDMS, 75-μm carboxen/PDMS (C/P) and carbowax-polyethylene glycol (C-PEG) fibers. Many peaks were eluted later in the TIC when we used a 7-μm PDMS fiber, as well as a C-PEG fiber (Figure 2a, top and bottom). This is because a 7-µm PDMS fiber favors non-polar and high-molecular-weight compounds or semi-volatile compounds, while a PEG fiber prefers to collect alcohols and polar compounds. On the other hand, a 75-μm C/P fiber can absorb gases and low-molecular-weight compounds, which are eluted early in the TIC (Figure 3a). The VOC profiles obtained using a 100-μm PDMS fiber were highly scattered in the TIC (Figure 3a). 

The HS-SPME-GC-TOF-MS analysis showed 437 peaks and their mass spectra. A total of 99 peaks were annotated as known VOCs or peaks with original IDs provided by VocBinBase [36] (Supplementary Table S1, S2). Principal component analysis (PCA) was applied to create a projection of multivariate data onto a space of lower dimensionality for visualization purposes (Figure 3b). The PCA score scatter plot showed clear separation in accordance with each fiber property. Often, SPME fibers coated with either 100-μm PDMS [42,53,56] or 65-μm-thick PDMS/divinylbenzene (DVB) [39,57] are used as a first choice, because the former prefers to absorb non-polar metabolites, while the latter can extract short-chain polar compounds, e.g., alcohols and amines. The PCA score scatter plot illustrates that samples obtained by using a 100-μm PDMS fiber and those obtained by using a 65-μm PDMS/DVB fiber were clustered very closely (Figure 3b). This suggests that the collection efficiency of these two fibers is probably very similar. We next evaluated the fiber specificity for the collected volatile substances in the HC of *Arabidopsis* seedlings by using the four representative fibers (75-μm C/P, 7-μm PDMS, 100-μm PDMS and C-PEG) that were chosen from the results of the PCA (Figure 3b). Among 99 annotated peaks, about half the peaks (47 peaks) could be commonly captured across the three fibers, unlike that in the case of those collected using the 100-μm PDMS fiber (Figure 3c). This result shows that the major VOCs collected from the developed cultivation system were likely to be less polar in terms of physicochemical properties. On the other hand, there were a few of the fiber-specific peaks (Table 1). In particular, a C-PEG fiber could attract relatively polar compounds, such as alcohols. We selected a 100-μm PDMS fiber as the first choice.

**Table 1:** Log_2_-fold changes infiber-specific peaks collected using the 75-μm C/P, 7-μm PDMS and C-PEG fibers, unlike that observed in the case of 100-μm PDMS fiber.

Compound annotation	log_2_-FC (C/P 75 *vs.* P 100)	FDR	log_2_-FC (P 7 *vs.* P 100)	FDR	log_2_-FC (C-PEG *vs.* P 100)	FDR
Heptane, 5-ethyl-2-methyl-	3.8	0.0000	n.s.		n.s.	
Dodecane, 4,6-dimethyl-	2.5	0.0046	n.s.		n.s.	
45514	n.s.		4.6	0.0139	n.s.	
1H-Indole, 1-methoxy-	n.s.		n.s.		4.5	0.0090
2-Undecanone	n.s.		n.s.		4.4	0.0000
Undecanal	n.s.		n.s.		3.7	0.0001
Biphenyl	n.s.		n.s.		6.6	0.0000
Dodecanal	n.s.		n.s.		3.9	0.0000
*n*-Tridecan-1-ol	n.s.		n.s.		5.4	0.0010

Significant, false discovery rate (FDR) of <0.05. Biological replicates, *n* = 4 per fiber. Abbreviations: log_2_-FC, log_2_-fold change; C/P 75, a 75-μm C/P fiber; P 7, a 7-μm PDMS fiber; C-PEG, a C-PEG fiber; P 100, a 100-μm PDMS fiber; n.s., not significant.

#### 2.2.3. Optimization of Incubation Conditions

**Figure 4:**
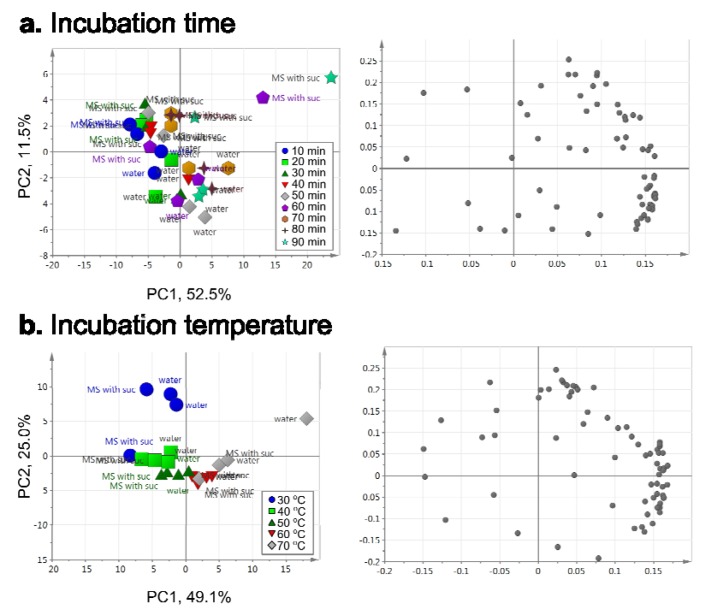
Evaluation of incubation time (a) and temperature (b) to optimize collection efficiency of volatiles in the HS vials containing *Arabidopsis* seedlings grown in water and MS conditions. Left, PCA score scatter plot. Each symbol represents an independent sample. Right, PCA loading scatter plot. The gray dot shows an annotated peak. Four independent samples (two samples per condition) were analyzed per incubation time or temperature.

We used a 100-μm PDMS fiber and evaluated collection efficiencies by changing incubation conditions, times and temperatures. The PCA was applied to visualize relationships between incubation time or temperature and collection efficiency of volatiles (Figure 4). We first evaluated incubation time to optimize the collection efficiency of volatiles. We fixed the incubation temperature at 60 °C and then performed HS-SPME-GC-TOF-MS analysis by setting the incubation times at 10, 20, 30, 40, 50, 60, 70, 80 or 90 min (Figure 4a). Most of the samples were clustered together on the PCA scatter plot; however, samples incubated at 60 and 90 min showed relatively low reproducibility (Figure 4a, left). The PCA loading scatter plot demonstrated that longer incubation times resulted in more peaks than shorter incubation times did (Figure 4a, right). As metabolomics requires high-throughput analysis with high reproducibility, we chose 20 min as the optimum time for the incubation of the SPME vials.

Next, we evaluated incubation temperature by setting it at 30 °C, 40 °C, 50 °C, 60 °C or 70 °C. The HS-VOCs were collected for 20 min by SPME with a 100-μm PDMS fiber. The PCA indicated that the largest source of variation was the difference in incubation temperature and, in particular, the difference between incubation at 30 °C and 70 °C (Figure 4b, left, PC1). Peaks were extracted at low and high incubation temperatures (Figure 4b, right), but samples incubated at 30 °C showed low reproducibility. On the other hand, the second source of variation could be explained by the differences between the samples incubated at 50 °C–70 °C, and those at 30 °C (Figure 4b, left, PC2) include aliphatic alcohols, aliphatic/aromatic aldehydes, esters, ethers, hydrocarbons, including alkanes, ketones and terpenes [39,50,53]. *Arabidopsis* and *Brassicaseae* species can emit isothiocyanates and nitriles [58]. These VOCs have high vapor pressures and low-to-medium water solubility. Since some terpenes are unstable at high temperature [59,60], incubations must be performed under mild conditions. Therefore, the incubation temperature was set at 60 °C. 

### 2.3. VOC Profiling by Using a Developed Workflow – A Case Example

We hypothesized that VOC profiles may differ when grown under the two nutrient conditions, because the biomass of the *Arabidopsis* seedlings grown in the MS condition was dramatically higher than that in water (Figure 1a,b). This implies that samples in water may be exposed to severe stress compared to those in MS. Using the optimized analytical conditions, we performed VOC profiling of *Arabidopsis* seedlings grown under the two conditions. 

**Figure 5:**
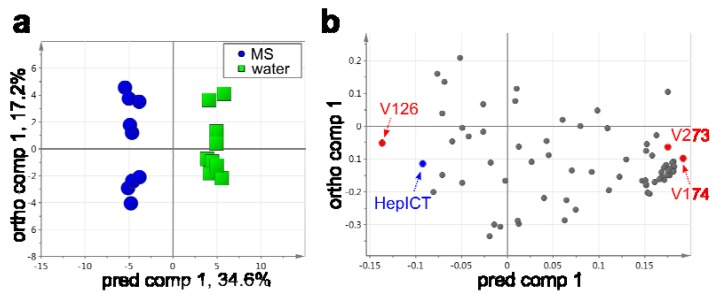
O2PLS-DA score scatter plot (*left*) and loading plot (*right*) of VOC profiles elicited from *Arabidopsis* seedlings grown under the two different conditions. Each symbol represents an independent sample in the score scatter plot and an independent annotated peak in the loading plot. The levels of VOCs annotated as V126, V174 and V273 emitted from the samples grown in water (biological replicates, *n* = 10) showed significant changes, when compared with those grown in MS (biological replicates, *n* = 10) (see Table 2). The metabolites were extracted according to the first-weight vector (w* 1) in addition to the 95% confidence intervals. A *p*-value (calculated by cross-validated predictive residuals, CV-ANOVA; *p* cv) of <0.01 for the model was defined as significant. Abbreviations: pred comp, predictive component; ortho comp orthogonal component; HepICT, 1-isothiocyanato-heptane.

We obtained 263 peaks and their mass spectra by performing HS-SPME-GC-TOF-MS analysis. A total of 69 peaks were annotated as known VOCs or peaks with VocBinBase IDs (Supplementary Table S1, S3). To find the VOCs that contributed to differences between the VOC profiles of samples harvested from MS and those from water, bidirectional orthogonal projection onto latent structure-discriminant analysis (O2PLS-DA) were performed [61,62]. In this case, O2PLS-DA can extract as much of the class-separating (MS *vs.* water) variation as possible. The O2PLS-DA score scatter plot of samples grown under the two conditions showed a clear separation (Figure 5a). For example, we could detect the peak annotated as 1-isothiocyanato-heptane (Figure 5b), which is produced from aliphatic glucosinolates [58,63]. When considering the biosynthesis of isothiocyanates in *Arabidopsis*, the isothiocyanate would contain a branched chain rather than an odd-numbered chain [64]. GC-high-resolution (HR)-TOF-MS analysis has great potential in more reliably estimating the structure of such a peak.

The 39 VOCs were predicted to be discriminative metabolites by the O2PLS-DA loadings in the first Y-predictive component. Of these, 37 peaks tended to increase in water (e.g., calamenen-10-one), while the levels of two peaks tended to increase when the samples were grown in MS (e.g., 3-Buten-2-one, 4-(2,6,6-trimethyl-1-cyclohexen-1-yl)-, (E)-). 

Subsequently, a *t*-test was applied to compare the two groups, MS *vs.* water (Table 2). The nine volatiles showed significant changes (false discovery rate (FDR) of <0.05). A significant decrease was observed in the level of the VOC, called V126, when compared to that from samples grown in MS. The peak V126 annotated as β-ionone is known to be a flavor VOC emitted from various plant tissues [65,66]. β-ionone is produced via the enzymatic cleavage of carotenoid by dioxygenase in tomatoes [65]. The significant decrease in the level of β-ionone grown in water may be linked to carotenoid breakdown, since only the existence of water is very severe for the survival of *Arabidopsis* seedlings (Figure 1a,b). For instance, carotenoid content decreases during aging in tobacco leaves [67], and it also decreases when *Arabidopsis* leaves are shifted from a nutrient-sufficient medium to water [68]. 

The levels of eight volatiles, including unknown peaks with a VocBinBase ID, showed significant increases when compared to those from samples in MS. The peak, V154, is probably a column-derived peak, such as that of cyclooctasiloxanes, while the peak, V143, was classified as that of terpenoids according to the mass spectrum. There are many articles regarding V130 [69,70,71,72] and V174 in plant tissues [73,74,75,76,77]. However, the current method could not identify the V174 peak, because of the compound’s orientation with respect to the benzene ring. 

**Table 2:** Significant changes in the levels of annotated volatiles.

Peak ID	Compound annotation	log_2_-FC (water * vs.* MS)	FDR
**V126**	3-Buten-2-one, 4-(2,6,6-trimethyl-1-cyclohexen-1-yl)-, (E)-	-4.1	0.0000
**V130**	Phenol, 2,4-bis(1,1-dimethylethyl)-	3.4	0.0000
**V143**	47409	1.3	0.0160
**V154**	45485	2.1	0.0126
**V172**	Hexadecanal	1.3	0.0239
**V173**	1,1'-Biphenyl, 2,2',5,5'-tetramethyl-	1.6	0.0069
**V174**	calamenen-10-one	1.7	0.0126
**V194**	Phthalic acid, decyl isobutyl ester	1.6	0.0307
**V273**	1-Phenanthrenecarboxylic acid, 1,2,3,4,4a,9,10,10a-octahydro-1,4a-dimethyl-7-(1-methylethyl)-, methyl ester, [1R-(1π4aπ10aπ]-	2.2	0.0261

The changes were represented as the log_2_- FC between the level of a volatile collected from samples grown in water and those grown in MS (significant, FDR < 0.05; biological replicates, *n* = 10 per condition).

By applying the developed method, we could discriminate between volatile compounds, including unknown peaks, from the two nutrient conditions, though they probably contained fiber- or column-derived compounds, such as biphenyl and silylated compounds. One of the current problems is in identifying the detected volatile peaks [78]. To accurately identify VOCs, authentic standards should be used in future studies. However, it is generally difficult to purchase authentic standards. As GC×GC-TOF-MS-based profiling can improve the separation of VOCs, the profiling technique is expected to provide high-resolution VOC profiles. Another possible way to estimate VOC structures is to use GC-HR-TOF-MS, because the technique can provide us a highly accurate *m/z* ratio with ultra-high resolution. Another big challenge of VOC profiling is to conduct absolute quantification of the detected peaks. We used one of the alkane standard peaks, *i.e.*, tridecane, as an internal standard (IS) in the study. However, it is insufficient, because we can only cover alkene-like compounds to predict partition coefficient and phase ratio of volatile compounds in a HS vial. The use of various types of compounds, as well as fully-labeled alkanes as ISs, can increase to cover physicochemical properties of detected VOCs as much as possible. The approach may help to improve data quality in terms of semi-quantification analysis of VOCs.

## 3. Experimental Section

### 3.1. Chemicals

The *n*-alkane standard solutions C8-C20 were purchased from Fluka Chemical (Tokyo, Japan). Other chemicals were purchased from Nacalai Tesque (Kyoto, Japan) or Wako Pure Chemical Industries (Osaka, Japan).

### 3.2. Plant Material

Wild-type *Arabidopsis thaliana* plants (accession Columbia (Col-0)) were used in this study. The plant seeds, which had been sterilized by sodium hypochlorite solution (Wako Pure Chemical Industries, Osaka, Japan), were stratified at 4 °C for 3 days. They were subsequently grown in MS medium (Wako Pure Chemical Industries) containing vitamins (Sigma-Aldrich, Tokyo, Japan; Lot, RNBB4051) with 0.8% agar and 1% sucrose at pH 5.8. Samples were exposed to fluorescent light under a 16-h light (51-μmol·m^−2^·s^−1^)/8-h dark cycle at 23 °C for 7 days in a growth chamber (MLR-350H; Sanyo, Osaka, Japan). Next, 20 seedlings were transferred into 20-ml HS vial (Supelco, Missouri, US) containing 5-mL of (i) sterile liquid culture of MS medium with 1% sucrose at pH 5.8 or (ii) sterilized Milli-Q water. The vials were closed with magnetic screw caps equipped with either Silicon/PTFE septa (AMR, Tokyo, Japan) or affixed with MilliSeals (EMD Millipore, Billerica, MA, USA). The seedlings were grown on a shaker (MMS-3010; Eyela, Tokyo, Japan) under a 16-h light 51-μmol·m^−2^·s^−1^)/8-h dark cycle at 23 °C for 7, 14 and 21 days. The shaker speed was set at 150 rpm during the cultivation. The HS vials were directly used for HS collection via SPME.

### 3.3. Headspace Collection and HS-SPME-GC-MS Analysis

We used two methods and assayed to collect the HS of *Arabidopsis* seedlings after a 7 day-incubation. To quench enzyme activity in *Arabidopsis*, we treated the seedlings in the following manner: (i) a set of HS vials wrapped in aluminum foil were incubated at 80 °C for 30 min in a windy oven (WFO-600 ND; Eyela, Tokyo, Japan) and (ii) 0.25-M EDTA-NaOH water solution (pH 7.5) was added into another set of vials to attain a final EDTA concentration of 50 mM. Solid CaCl_2_ was then immediately added to a final concentration of 5 M [39]. Next, 10-μL of *n*-alkane standard solutions C8-20 (0.8 mg/l) was added to each vial as an internal standard. The vials were closed with magnetic screw-caps and then sonicated (US-108; NSD, Suwa, Japan) at a frequency of 38-Hz for 5 min. Vials without any treatment were prepared and used as controls. 

Nine types of SPME fibers were purchased from Supelco (Supelco, PA, USA). A SPME fiber coated with a 100-μm-thick layer of polydimethylsiloxane (PDMS) metal alloy (100-μm PDMS) was finally chosen for VOC profiling (see Results and Discussion section). SPME fibers were coated with (i) 30-μm- or (ii) 7-μm-thick layers of PDMS-fused silica (FS) fiber/stainless steel (SS), (iii) 75-μm-thick layer of carboxen/polydimethylsiloxane (CAR/PDMS) FS/SS, (iv) 85-μm-thick layer of CAR/PDMS StableFlex (SF) fiber/SS, (v) 65–μm-thick layer of PDMS/divinylbenzene (DVB) SF/SS, (vi) 50/30-μm-thick layer of DVB/CAR on PDMS (DVB/CAR/PDMS) SF/SS, (vii) 85-μm-thick layer of polyacrylate (PA 85) or (viii) carbowax-polyethylene glycol (C-PEG) for HS collection. Before the analysis, all fibers were conditioned at the appropriate conditioning temperature and time as shown in Table 3. Each fiber was exposed to the vial headspace for 20 min at 60 °C with continuous agitation.

After HS collection, the volatiles were thermally desorbed in splitless mode on a CTC CombiPAL autosampler (CTC analytics, Zwingen, Switzerland) connected to an Agilent 6890N gas chromatograph (Agilent Technologies, Wilmington, USA) for 0.1 min at the appropriate inlet temperature, as shown below. Each fiber was baked for 5 min by applying the appropriate conditioning temperature (see below).

**Table 3:** Conditioning temperature and time for the SPME fibers used in the study.

SPME fiber	Conditioning temperature ( °C)	Conditioning time (h)	Inlet temperature ( °C)
100-μm PDMS	250	0.5	220
30-μm PDMS	250	0.5	220
7-μm PDMS	320	0.5	220
85-μm polyacrylate	280	1	220
60-μm PEG	240	0.5	220
75-μm CAR/PDMS	300	1	280
85-μm CAR/PDMS	300	1	280
65-μm PDMS/DVB	250	0.5	250
50/30-μm DVB/CAR/PDMS	270	0.5	250

The capillary column used for the analysis was a 30-m×0.25-mm inner diameter fused-silica capillary column with a chemically bound 0.25-μL film Rxi-5 Sil MS stationary phase (RESTEK, Bellefonte, USA) with a tandem connection to a fused silica tube (1 m, 0.15 mm). A mass spectrometer column change interface (ms NoVent-J; SGE, Yokohama, Japan) was used to prevent air and water from entering the MS during column change over. Helium was used as the carrier gas at a constant flow rate of 1.0 ml/min. The temperature program started with a 2 min isothermal step at 50 °C, followed by temperature ramping at 15 °C to a final temperature of 260 °C, which was then maintained for 2 min. The transfer line to the mass spectrometer was set to 250 °C. The TOF mass spectrometer was a Pegasus 4D MS system (Leco, MI, USA) with an EI source set to 200 °C. The acceleration voltage was turned on after a solvent delay of 200 s. Mass spectra were monitored with an acquisition rate of 30 spectra/s and over a mass-to-charge ratio range of *m*/*z* = 30–550.

### 3.4. Data Processing

Non-processed MS data from GC-TOF-MS analysis were exported in NetCDF format, which was generated by the chromatography processing and mass spectral deconvolution software (Leco ChromaTOF version 2.32; LECO, MI, USA), to MATLAB 7.0 and MATLAB2011b (Mathworks, MA, USA), where all data pretreatment procedures, such as smoothing, alignment, time-window setting and hierarchical multivariate curve resolution (H-MCR), were carried out [79]. The resolved mass spectra were matched against the reference mass spectra by using the NIST mass spectral search program from the NIST/EPA/NIH mass spectral library (Data Version: NIST 08, Software Version 2.0) [34], Terpenoid library, Identification of Essential Oil Components by Gas Chromatography/Mass Spectrometry (the 4th Edition) commercial library [35] and VocBinbase [36]. Our custom software was also used for peak annotation written in Java. The metabolites were defined as annotated metabolites by comparison with RI values calculated by alkane standard mixtures (C8–20) and mass spectra from these three libraries. Peaks that showed a match factor of ≥800 in the mass spectral library were selected for annotation. Data were normalized by dividing each peak area by the peak area of *n*-tridecane and metabolite identifiers were organized using MetMask [80].

### 3.5. Data Analysis

The two data matrices (observations: biological replicates, variables: annotated peaks) were used for statistical analyses, respectively (see Supplementary Tables S1–3). Multivariate analysis was performed using the SIMCA-P+13.0 software (Umetrics AB, Umeå, Sweden), with log_10_ transformation and unit variance scaling. Metabolite profile data were log_2_ transformed and then statistically analyzed using the LIMMA package [81], which includes false discovery rate (FDR) correction for multiple testing (Benjamini and Hochberg, 1995) in the R environment for statistical computing (version 2.14.2). Analysis of variance testing of cross-validated predictive residuals (CV-ANOVA) was used as the significance test for assessing the reliability of O2PLS-DA models (significant, *p* cv < 0.01) [82]. 

## 4. Conclusions

VOC profiling using HP-SPME-GC-TOF-MS can distinguish between profiles with respect to each nutrient condition. More VOCs were expelled into the HS when *Arabidopsis* seedlings were exposed to conditions of limited nutrient supply, and the biomass of the seedlings showed severe growth reduction. Since we could easily change the nutrition conditions to grow the seedlings, we believe that the developed method will work well in investigating the assimilation of inorganic nutrients and production of VOCs, not only in *Arabidopsis*, but also in other plants. In addition, we can conduct metabolite profiling of *Arabidopsis* seedlings themselves grown using the cultivation method. This suggests a great potential for the integrated analysis of VOC profile data and metabolite profile data, not only to capture the changes in the levels of VOCs in plants grown under various environmental conditions, but also to understand the relationships among nutrition, biomass and metabolic networks involving primary and volatile metabolites.

## Supplementary Files

Supplementary File 1 Supplementary Tables (XLSX, 20 KB)
